# Combining factorial and multi-arm multi-stage platform designs to evaluate multiple interventions efficiently

**DOI:** 10.1177/17407745221093577

**Published:** 2022-05-17

**Authors:** Ian R White, Babak Choodari-Oskooei, Matthew R Sydes, Brennan C Kahan, Leanne McCabe, Anna Turkova, Hanif Esmail, Diana M Gibb, Deborah Ford

**Affiliations:** MRC Clinical Trials Unit at UCL, London, UK

**Keywords:** Platform trials, adaptive trial designs, factorial trial, multi-arm multi-stage

## Abstract

**Background:**

Factorial designs and multi-arm multi-stage (MAMS) platform designs have many advantages, but the practical advantages and disadvantages of combining the two designs have not been explored.

**Methods:**

We propose practical methods for a combined design within the platform trial paradigm where some interventions are not expected to interact and could be given together.

**Results:**

We describe the combined design and suggest diagrams that can be used to represent it. Many properties are common both to standard factorial designs, including the need to consider interactions between interventions and the impact of intervention efficacy on power of other comparisons, and to standard multi-arm multi-stage designs, including the need to pre-specify procedures for starting and stopping intervention comparisons. We also identify some specific features of the factorial-MAMS design: timing of interim and final analyses should be determined by calendar time or total observed events; some non-factorial modifications may be useful; eligibility criteria should be broad enough to include any patient eligible for any part of the randomisation; stratified randomisation may conveniently be performed sequentially; and analysis requires special care to use only concurrent controls.

**Conclusion:**

A combined factorial-MAMS design can combine the efficiencies of factorial trials and multi-arm multi-stage platform trials. It allows us to address multiple research questions under one protocol and to test multiple new treatment options, which is particularly important when facing a new emergent infection such as COVID-19.

## Background/aims

Randomised controlled trials (RCTs) give high-quality evidence of the effects of clinical interventions, but they are complex, expensive and time consuming. Factorial designs can improve the efficiency of RCTs by allowing two or more randomised evaluations independently in the same sample of patients without substantial increase of sample size; they can also allow testing for interactions or synergy between interventions.^1^ Multi-arm multi-stage (MAMS) designs can also improve efficiency: the multi-arm element allows simultaneous assessment of a number of research interventions, usually compared with the same control group; the multi-stage element speeds up the evaluation process and reduces its expense by allowing recruitment to insufficiently active research approaches to be ended early. Platform designs further speed up the evaluation process by allowing new interventions to be introduced for assessment during the course of the trial.^2^ Here, we use the term MAMS to refer to multi-arm multi-stage platform designs.

Given their advantages, it may be useful to combine factorial and MAMS design features. This has not been explored in detail in the literature. Generally, they have been regarded as alternative designs^3^ or as competitors rather than as elements to combine.^4^ One MAMS trial explicitly rejected a factorial design because the assumptions of a factorial design were not met.^5^ A notable exception is the RECOVERY trial of treatments for COVID-19 which started as a multi-arm adaptive platform trial but soon added in tocilizumab as a factorial randomisation in a subgroup; further factorial randomisations followed.^6^ A randomised embedded multifactorial adaptive platform also combines factorial and adaptive designs, with the additional inclusion of precision medicine ideas, response-adaptive randomisation and Bayesian analysis.^7,8^

This article outlines some general methodological considerations in combining the two designs into a ‘factorial-MAMS’ design, focusing on a late-phase trial setting with a binary or time-to-event outcome. The work was initially motivated by a proposed trial of treatments in the COVID19 pandemic, where evidence accrues quickly.^9^ However, this article is of broader relevance. Our aims are to (1) introduce the factorial-MAMS trial design and suggest some visual representations, (2) identify some difficulties in design, conduct and analysis that arise when factorial and MAMS elements are combined, (3) suggest how these difficulties should be addressed and (4) offer guidance on when such a trial may be useful. We focus on the underlying principles and ideas rather than the statistical details of the design.

## Methods

### Factorial designs

We consider a factorial design to be one where two or more randomisations occur concurrently in the same patients, and where the analysis reflects this structure. A factorial design may be appropriate where two or more interventions (or other aspects of management) are to be evaluated, the interventions may be combined, and the receipt of one intervention is unlikely to affect the benefit received from another intervention.^10^ For example, in a two-by-two factorial design to evaluate two new interventions A1 and B1, a first randomisation might be to add A1 or A0 to standard care, where A0 is the appropriate control or placebo, and the second randomisation might also add B1 or a corresponding control or placebo B0. Patients would therefore be randomised to add A0+B0, A0+B1, A1+B0 or A1+B1. We call these four groups the arms of the trial. More complex factorial trials can also be used, for example a 3 × 2 × 2 factorial.^11^

A factorial (or ‘at the margins’) analysis compares all patients randomised to A1 with all those randomised to A0, that is A1+B0 plus A1+B1 compared with A0+B0 plus A0+B1 (and similarly for B1 vs B0). Where the target intervention effects for A1 and B1 are the same and there is no interaction between A1 and B1, the sample size for evaluating A1 can be the same as in a simple two-arm trial, alongside an equal ability to evaluate B1. Such a trial is sometimes described as ‘two trials for the price of one’.^12^ However, if B1 is an effective intervention then the number of events in a factorial trial is less than in a simple two-arm trial without B1, and evaluating A1 requires more patients or longer follow-up.

The possibility of an interaction complicates factorial trials in a number of ways. Usually the interaction is expected to reduce the benefit of the combined treatment, and this reduces the power of the factorial analysis to detect treatment effects.^13^ The estimand targeted by the factorial analysis is a comparison of A1 with A0 in a population which experiences randomisation to B1 or B0, but the typical estimand of interest^14^ is the comparison of intervention A1 with A0 in the absence of intervention B1, or (if B1 is clearly effective) in the presence of B1; the factorial estimator will be biased for both of these estimands differ in the presence of interaction.

These problems are hard to resolve because factorial designs are typically insufficiently powered to explore interactions and arm-vs-arm comparisons.^1^ If interaction is anticipated then other statistical methods and a larger sample size are required: possibilities include structured comparisons between the four arms^15,16^ or a MAMS approach where each treatment combination is taken as a separate treatment.^4,5^

In the remainder of this article, we assume that interaction between different factors of the factorial design is unlikely and that the main analysis will be factorial.

### MAMS platform designs

A MAMS design compares one or more interventions with a control intervention in a parallel fashion.^17^ For example, interventions A and B might be separately compared with control by randomising to three arms: A, B or control. As outcome data accrue, one or more planned interim analyses may lead to comparisons being stopped for lack-of-benefit, because the interventions are unlikely to be beneficial. These interim analyses may use an intermediate outcome measure that is of lower clinical importance, but more information-rich, than the definitive trial outcome measure and which investigators can be confident will be improved by any effective intervention.^18^ This intermediate outcome measure can be on the causal pathway rather than a true surrogate. Interventions may also be stopped with early evidence of efficacy. Guidelines for stopping interventions should be pre-defined.^19^ Analysis uses standard methods, but care must be taken to control the type-1 error rate.^20^ Adding interventions during the course of the trial can be anticipated in a platform protocol, sometimes referred to as a ‘living protocol’ or a ‘master protocol’.^21^

MAMS designs have several advantages. They can evaluate several primary hypotheses or interventions within the same protocol, giving a greater chance of identifying an effective new intervention than in any two-arm trial.^22^ They can seamlessly span phases II and III, and so require fewer patients than separate phase II and III trials. They allow major adaptations, such as ceasing randomisation to a research arm or introducing new research arms, and so fewer patients tend to be exposed to ineffective or harmful interventions as the trial progresses. Finally, patients in MAMS designs are more likely to receive active interventions, which may help recruitment.

### A proposed factorial-MAMS design

This work is motivated by a trial, proposed in mid-2020, of treatments for COVID-19 infection in non-hospitalised patients in Africa. Ultimately, the trial was not funded and so this implementation of this new design was not finalised. Our discussion avoids the details of the specific treatments proposed and considers a simplified trial that starts with a 2 × 2 factorial design, comprising two two-way randomisations: one comparing an antiviral agent with standard of care, and one comparing an immunomodulator with standard of care. The no-interaction assumption was considered reasonable here. COVID-19 treatments were developing fast, so the trial design anticipated adaptations being required – both adding and dropping treatments.

## Results

### Illustrations of a factorial-MAMS design

**Figure 1.**
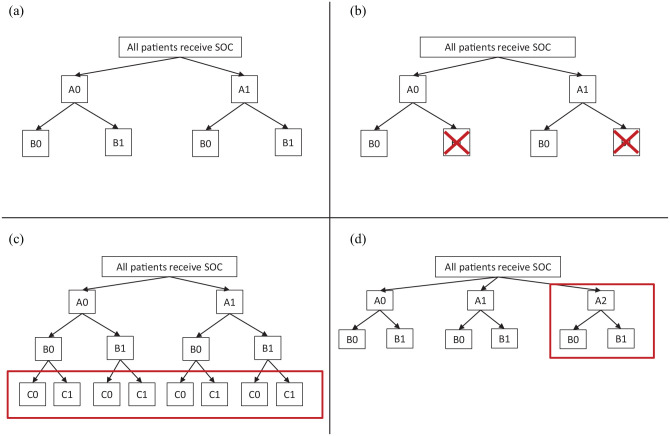
Randomisation possibilities in an illustrative factorial-MAMS trial: (a) initial design before any adaptation, (b) adapted design after stopping treatment B1, (c) adapted design after adding treatment C1 and (d) adapted design after adding treatment A2.

Figure 1(a) illustrates the initial 2 × 2 factorial design. We show the interventions generically as A0, A1 for randomisation A and B0, B1 for randomisation B. In the illustrative example, A1 is the antiviral agent, B1 is the immunomodulator, and A0 and B0 are the corresponding control treatments, so standard of care is A0+B0.

The other three panels of Figure 1 show ways in which the trial could be adapted in the light of accruing trial data and/or external evidence. Criteria for making these adaptations are discussed in later sections. In Figure 1(b), intervention B1 is stopped for lack-of-benefit. Subsequent patients are still randomised to A0 or A1 but all receive B0 (if B0 is an active intervention) or no intervention (otherwise). In Figure 1(c), a third intervention is added which can be combined with any of the other trial interventions. Therefore, it is called C1, and a third randomisation to C1 or a corresponding control C0 is added. In Figure 1(d), a different third intervention is added which can be combined with B0 and B1 but for clinical or practical reasons cannot be combined with A1 (e.g. it is an alternative antiviral agent). Therefore, it is added into randomisation A and is called A2. Table 1 shows an alternative illustration of the same adaptations.

**Table 1.** Example adaptations of a factorial-MAMS trial over time.

Panel of Figure 2	Adaptation	Randomisation A	Randomisation B	Randomisation C	Combinations active
Figure 2(a)	Initial design	A1 vs A0	B1 vs B0	–	A0+B0; A1+B0; A0+B1; A1+B1
Figure 2(b)	Adapted designafter stoppingintervention B1	A1 vs A0	–	–	A0; A1
Figure 2(c)	Adapted design afteradding intervention C1	A1 vs A0	B1 vs B0	C1 vs C0	A0+B0+C0;A0+B0+C1;A1+B0+C0;A1+B0+C1;A0+B1+C0;A0+B1+C1;A1+B1+C0;A1+B1+C1
Figure 2(d)	Adapted design afteradding intervention A2	A1 vsA2 vs A0	B1 vs B0	–	A0+B0; A1+B0;A2+B0; A0+B1;A1+B1;A2+B1

**Figure 2.**
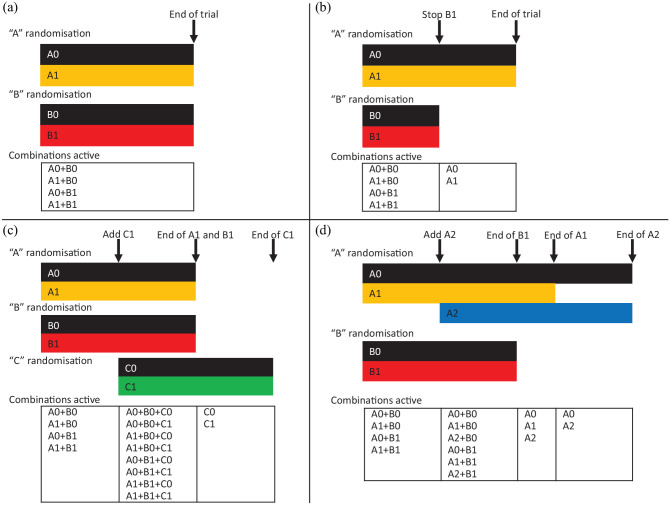
Trial evolution in an illustrative factorial-MAMS trial: (a) original design is not adapted, (b) original design is modified by stopping treatment B1, (c) original design is modified by adding treatment C1 and (d) original design is modified by adding treatment A2.

Figure 2 shows how the trial may develop over time as adaptations occur. For this figure, we make the simplifying assumption that all randomisations use equal allocation and that the sample size needed for each comparison is the same; in practice, one would consider increasing the sample size to allow for efficacious interventions in other comparisons, as discussed in the Sample Size Implications section. We assume for simplicity that information (events or binary outcomes) accrues at a constant rate and a single interim analysis is performed when half the planned information has accrued. Figure 2(a) shows the case when the trial is not adapted. Time moves from left to right and both comparisons (A1 vs A0 and B1 vs B0) end at the initially planned end of the trial. Figure 2(b) shows the case where intervention B1 is stopped at the interim analysis. Figure 2(c) shows the case where a C randomisation is introduced. Because the C comparison starts late, recruitment to the C comparison must extend beyond the initially planned end of the trial, but because it is added factorially, it does not affect completing recruitment to the A and B comparisons. Figure 2(d) shows the case where a third intervention is added to randomisation A. After this point, information accrues more slowly to each of the A comparisons, assuming the same accrual rate. Therefore, three-way randomisation must extend beyond the initially planned end of the trial, and randomisation to A2 or A0 must extend beyond that. Recruitment to the B comparison, however, is unaffected by the adaptation and still ends at the planned end of the trial.

Table 2 shows an alternative representation of the initial design and the adapted design in Figure 2(d). For illustrative purposes, it assumes that each comparison requires 3000 patients and that this number is unaffected by multiplicity corrections. The original design ends after data on the planned 3000 patients have been collected. The adapted trial can report the B1 versus B0 comparison at this point, but it must recruit and collect data on 750 more patients before reporting the A1 versus A0 comparison, and a further 1500 before reporting the A2 versus A0 comparison.

**Table 2.** Progress of the factorial-MAMS trial in Figure 2(d), when an intervention A2 is added partly factorially after stage 1. Each comparison is assumed to require 3000 patients.

Stage	Design	Patients randomised	Patients contributing to comparison		
			A1 vs A0	B1 vs B0	A2 vs A0
*Unadapted design*					
1	(A1 vs A0) × (B1 vs B0)	1500	1500	1500	–
2	(A1 vs A0) × (B1 vs B0)	1500	1500	1500	–
Total		3000	3000	3000	–
*Design adapted by adding A2 after Stage 1*					
1	(A1 vs A0) × (B1 vs B0)	1500	1500	1500	–
2	(A1 vs A2 vs A0) × (B1 vs B0)	1500	1000	1500	1000
3	(A1 vs A2 vs A0)	750	500	–	500
4	(A2 vs A0)	1500	–	–	1500
Total		5250	3000	3000	3000

### Design adaptations

In this section, we give more detail on the timing and implementation of the adaptations described above and their advantages and disadvantages.

#### Timing of interim analysis

In MAMS designs with binary outcomes, interim analyses are usually scheduled when a fixed number of patient outcomes have been observed. In MAMS designs with time-to-event outcomes, however, timing of interim analyses is usually based on observed outcome events in the control arm^23^ or across all arms.^24^ The former can lead to slightly worse type-1 error control and can postpone decision when treatment is inefficacious,^24^ but we advocate it for two reasons. First, a total event rate better or worse than anticipated may suggest that the treatment effect is similarly better or worse than anticipated, which risks unblinding the trialists to the trial results. Second, with more than one experimental arm, timing based on control arm events allows the convenience of performing interim analyses for all comparisons simultaneously. This is an area for further research.^23^

Unlike in a MAMS, where each comparison has the same control arm, each comparison in a factorial design has a different control. Revealing the number of events in each of these controls would provide information about intervention effects. For example, in the scenario described in Table 2, if the A1 versus A0 comparison has 70 control arm events (comprising the A0+B0 and A0+B1 arms) and the B1 versus B0 comparison has 50 control arm events (comprising the A0+B0 and A1+B0 arms), then it is clear that A1+B0 is performing better than A0+B1, suggesting that either A1 is beneficial or B1 is harmful. For a combined factorial-MAMS design, we therefore advocate that the timing of interim analyses is either driven by the number of events in all arms combined, or is fixed in advance.

#### Stopping interventions for lack-of-benefit

An intervention is stopped for lack-of-benefit if it is unlikely to be found to be sufficiently efficacious in the trial to impact practice. Standard MAMS software can be used to devise a suitable stopping guideline^25^ as shown in the Supplemental material. Stopping would end randomisation to the intervention and could also end trial use of the intervention and/or follow-up of patients allocated to the intervention.

In a two-arm multi-stage design, the main arguments for stopping for lack-of-benefit are limiting the use of an ineffective intervention and limiting the duration of a negative trial. In a two-way randomisation in a factorial-MAMS trial, the former is relevant, but the duration of the trial is not reduced if other comparisons continue. In a three-or-more-way randomisation, however, the duration of the trial can be reduced by stopping an intervention, because subsequent patients can be allocated to the other interventions. For example, in Table 2, if recruitment to A1 had been stopped after stage 1, subsequent patients would be randomised to A2 versus A0 and the total sample size reduced from 5250 to 4500.

#### Stopping interventions for efficacy

Assessment of an intervention is stopped for efficacy in a MAMS design if there is convincing evidence of efficacy (benefit) on the definitive (not intermediate) outcome. When recruitment to any of the interventions may be stopped for efficacy, it is crucial to control the type-1 error rate. This is generally done by careful specification of interim stopping rules (15) including Haybittle-Peto, O’Brien-Fleming, or those based on an alpha-spending function (21).

After stopping any intervention (say, A1) for efficacy, standard of care in routine practice is likely to be modified to recommend or mandate use of A1 and the trial should reflect this. Recruitment to the other comparisons B, C and so on should continue, with the only modification being recognition that the wider use of A1 reduces the event rate for the other comparisons and hence may require larger sample sizes (for a binary outcome) or longer follow-up (for time-to-event outcomes). The consequences for other treatments in the A comparison are more complicated. A classic MAMS trial would end randomisation A at this point. However, clinical questions might remain about the other treatments (say, A2): their efficacy compared with A0; their non-inferiority to A1 (which would not have been powered originally), if they are (say) less expensive than A1; or their efficacy in combination with A1. Furthermore, follow-up will yield further data relevant to the first two questions, except in settings where A0 and A2 patients may be immediately offered A1 (e.g. a repurposed treatment in a chronic disease). It may be appropriate to address one of these clinical questions more fully as a new comparison within a modification of randomisation A. This raises problems which need to be addressed and are the subject of future work: whether to pause randomisation A while the new comparison is being prepared; whether to include the earlier comparative data in the analysis, and if so how to control type-1 error; and what sample size is required.

#### Adding interventions for assessment

An intervention may be added for evaluation in a factorial-MAMS design, as in a standard MAMS design, if there is new external evidence of benefit.^26^ This brings the advantage that a promising intervention may be evaluated much more quickly than by setting up a new trial.^27^ Furthermore, if multiple competing interventions (i.e. in the same randomisation) show benefit, the design may also be able to estimate their relative benefits from the head-to-head data.^28^

One disadvantage, shared with a standard MAMS design, arises if the trial needs to strongly control the family-wise type-1 error rate. Then adding a new intervention arm reduces the nominal significance level for existing comparisons and hence reduces their family-wise power.

Several disadvantages are shared with the factorial design. If the added intervention is effective and the outcome measure is binary or time-to-event, then there will be fewer events for existing comparisons, so their (pairwise) power will be reduced. If the new intervention is added to an existing randomisation, then patients allocated to the new intervention will not contribute to other comparisons in the same randomisation, and the timeline to recruit for these other comparisons should be extended, as for comparison A1 versus A0 in Figure 2(d). Trialists might, therefore, consider delaying adding a new intervention comparison until recruitment to existing arms is complete.

Disadvantages unique to the factorial-MAMS design involve timing of analyses. Adding intervention A2, as in Figure 2(d), means that the timing of the final results for A1 versus A0 needs to be moved later, and final results for B1 versus B0, A1 versus A0 and A2 versus A1 must all be reported at different times. Similarly, the optimal timing for interim analyses may vary for the different comparisons, so timing meetings of the Independent Data Monitoring Committee will involve compromise.

#### Estimands

Each of the adaptations above affects the estimand targeted by a factorial analysis of the other interventions in the presence of possible interactions. The unadapted design estimates the intervention effect in a population of whom some fraction receives each other factorial intervention. Stopping delivery of one of the other factorial interventions modifies this fraction, and adding a new factorial intervention introduces a fraction of the new intervention into the mix.

For instance, consider Figure 2(c). The estimand targeted by a factorial analysis of A1 versus A0 is an average of six different estimands: the effect of A1 versus A0 when patients are allocated to B0 alone, B1 alone, B0 and C0, B0 and C1, B1 and C0, or B1 and C1. However, if the no-interaction assumption is true, then this average estimand is equal to the estimand of clinical interest.

### Design considerations

#### Eligibility

It is reasonable to recruit certain patients to just some of the randomisations, or (in a randomisation to more than two interventions) to randomise them between a reduced set of interventions.^29,30^ For example, if intervention A1 was contra-indicated in pregnancy, then pregnant women would receive intervention A0 and could still enter randomisation B. Potential patients must, therefore, be screened before randomisation for eligibility for each intervention. Unwillingness to be randomised can also be accommodated: for example, a patient might request intervention A0 but enter randomisation B. Trial eligibility criteria should therefore be written broadly.

#### Stratified randomisation

Stratified randomisation aims to balance sample sizes and selected covariates, termed stratification factors, between randomised groups.^31^ In a factorial design, it is natural also to stratify each randomisation by the other allocations. This may be achieved by simultaneous randomisation of all interventions. In the running example, assuming block randomisation, we would form blocks of size *b* within strata, where *b* is a (typically varying) multiple of the number of arms (4), and then ensure that each block is assigned equally to the four arms (A0+B0, A1+B0, A0+B1, A1+B1). Imbalances in sample sizes and stratification factors between randomised groups then occur only due to some blocks being incomplete. The expected degree of imbalance increases as the number of strata increases.

Stratified randomisation may also be achieved by *sequential randomisations*, each stratified by stratification factors and the previous randomisations. Here, later randomisations have more strata and hence more potential for incomplete blocks and greater expected imbalance.

The inherent complexity of a factorial-MAMS trial makes it desirable to keep the randomisation as flexible as possible. Sequential randomisations can be handled separately and modified separately as interventions are added or dropped. We, therefore, focus on sequential randomisations and consider two complications which occur in a factorial-MAMS trial.

First, we need to handle patients who are not eligible for, or do not consent to, certain randomisations. In subsequent randomisations, it is then not clear which stratum they fall in. We suggest adding a ‘not randomised’ level to each stratification factor that is a previous randomisation. Thus randomisation A in the original design in Figure 1(a) would have three strata, ‘randomised to A0’, ‘randomised to A1’ and ‘not in randomisation A’, for each level of other stratification factors.

Second, we need to modify the randomisation scheme when an intervention is added or dropped. Approaches are described in Table 3.

**Table 3.** How stratified sequential randomisation may be adapted when an intervention is added to or dropped from a particular randomisation.

Trial adaptation	Approach	Details for the randomisation at which intervention is added/dropped	Details for later randomisations
Add new intervention as a new randomisation	Add as the last in the sequence of randomisations to avoid disrupting programming for previous randomisations	The number of strata for this new randomisation should be checked and (if it is large) alternatives to block randomisation should be considered	Not applicable since this is the last randomisation
Add intervention to an existing randomisation	This randomisation continues in modified form	Existing blocks for this randomisation should be closed and new blocks used for future patients	Create new strata for patients previously allocated to the new intervention. Assign other patients in the existing strata and to the existing blocks
Drop intervention from a two-way randomisation	This randomisation is discontinued	N/A	Use the strata for “not randomised” in the discontinued randomisation
Drop intervention from a three-way or more randomisation	This randomisation continues in modified form	Existing blocks for this randomisation should be closed and new blocks used for future patients	Continue assigning patients to existing blocks in the on-going strata

Minimisation is an alternative if stratified randomisation would give too many strata. This would allow any imbalance that exists for continuing interventions at the point of adaptation to be corrected after adaptation.

#### Sample size implications

An initial approach to sample size calculation should consider each randomisation separately and use standard methods for MAMS trials.^19,25,32^ Sample sizes need not be the same for each comparison, implying recruitment may finish at different times and/or separate final analyses. If control of the family-wise error rate is required, including in the case of adding arms, then this should be taken into account in the calculation, leading to larger sample sizes or longer durations.^21,33^ Controlling family-wise error rate requires consideration of the number of interventions at the trial start and the number that may be added. If only pairwise control of the type-1 error rate is required, the main issue is early stopping for efficacy.

The factorial element complicates considering each randomisation separately, since the event rate will be lowered if an intervention in another randomisation is effective. In a simple factorial trial, this could be done by exploring the loss of power due to other interventions being effective, compared with the base case of no other effective interventions; however, as the number of other interventions increases it becomes sensible to assume that one or more of them is effective. Similarly, changing to a more efficacious standard of care (perhaps a successful trial intervention) reduces the event rate and requires a longer trial duration for the same number of events. An example sample size calculation is given in the Supplemental material.

### Trial conduct considerations

Procedures for adding and stopping interventions should be clearly specified in the trial protocol and should be clear to regulatory authorities and ethics committees.^34^ Statistical guidelines to inform decisions for stopping interventions should be specified in the protocol and statistical analysis plan and agreed by the oversight committees.

Operational aspects are as in other platform protocols.^27,35,36^ Difficulties may relate to obtaining funding, ethical approval and regulatory approval. Patient and public involvement has shown that testing many interventions at once in MAMS trials is popular with patients,^22^ and we expect the same to be true of the hybrid design. Changing standard of care based on initial results could present problems and could happen at different speeds in different sites.

### Analysis issues

The key principle in the analysis of any complex randomised trial is to compare patients randomised to intervention with their concurrent controls. We describe evaluating intervention A1 in the interim or final analysis of a factorial-MAMS trial. The preferred analysis compares those randomised to A1 with those contemporaneously randomised to A0, regardless of which other randomisations they underwent (B, C etc.) and regardless of what interventions they were assigned to in those other randomisations. Note that patients allocated to A0 or A1 other than through randomisation are excluded from this comparison. We must also restrict the comparison to patients who could have been randomised to either A0 or A1: for example, in Figure 2(d), we exclude patients randomised to A0 after A1 had stopped.

Typically, each comparison is performed separately, but they could all be performed in a single statistical model.^8^ The model includes one dummy variable for each intervention, other than the control intervention, in each randomisation. To preserve the principle of concurrent control, it must also control for trial stage, where each stage is defined by a change in the randomisation scheme. Stage boundaries are marked as vertical arrows in Figure 2. Patient ineligibility for particular randomisations or interventions introduces further complications discussed in the Supplemental material.

Analysis of a factorial design usually includes exploring the interaction between the intervention of interest and subgroups defined by the other randomised interventions. This remains sensible in a factorial-MAMS design, but stopping and starting interventions may make some of these subgroups very small, so power for the interaction tests may be even lower than in a standard factorial design. For example, in the lower right panel of Figure 2, we would compare B1 with B0 overall and in the three subgroups receiving A0, A1 and A2, but the subgroup receiving A2 would be only half the size of the other subgroups.

### Extensions

We have described designs with binary and time-to-event outcomes, equal allocation ratios and with each comparison requiring the same sample size. Continuous outcomes would raise similar issues, except that, assuming no interactions, one effective intervention would not be expected to reduce the power for other comparisons since the power does not depend on an event rate. Allocating more patients to control than to any experimental arm has benefits which become important as the number of interventions increases.^5^ Comparisons with different target intervention effects would require different sample sizes.^21^

## Conclusion

The factorial-MAMS design is useful in a setting where multiple interventions need to be evaluated and some of them could reasonably be combined without the expectation of interaction. It has all the complexities of the factorial and the MAMS design and some extra complexities of its own, in particular that the use of multiple interventions increases the risk that some interventions will interact. However, if used well, the factorial-MAMS design has the potential to evaluate multiple interventions in a faster way than any alternative design. Our proposal represents a good balance of an efficient and flexible design.

## Supplemental Material

sj-pdf-1-ctj-10.1177_17407745221093577 – Supplemental material for Combining factorial and multi-arm multi-stage platform designs to evaluate multiple interventions efficiently Supplemental material, sj-pdf-1-ctj-10.1177_17407745221093577 for Combining factorial and multi-arm multi-stage platform designs to evaluate multiple interventions efficiently by Ian R White, Babak Choodari-Oskooei, Matthew R Sydes, Brennan C Kahan, Leanne McCabe, Anna Turkova, Hanif Esmail, Diana M Gibb and Deborah Ford in Clinical Trials
