# Supplementary material for: Combining factorial and multi-arm multi-stage platform designs to evaluate multiple interventions efficiently
Source: Clin Trials. 2022 May 17;19(4):432–41. doi: 10.1177/17407745221093577 (PMC9373200; doi:10.1177/17407745221093577)
Supplement: sj-pdf-1-ctj-10.1177_17407745221093577 – Supplemental material for Combining factorial and multi-arm multi-stage platform designs to evaluate multiple interventions efficiently [file sj-pdf-1-ctj-10.1177_17407745221093577.pdf]

Supplementary materials for "Combining factorial and MAMS platform designs to evaluate multiple interventions efficiently"

1. *Design: example of a sample size calculation*

1.1. Two-arm conventional design

We consider the proposed trial evaluating treatment of COVID-19 infection in non-hospitalised patients in Africa, using the initial design as in Figure 2A. Equal allocation was used in each randomisation. The outcome was 28-day mortality and was expected to occur in $\pi_0 = 17.9\%$ of patients randomised to control. To give round answers, we change this to $\pi_0 = 16.73\%$. Detecting a $\delta = 25\%$ reduction to $\pi_1 = 12.55\%$ with 90% power requires a sample size of 3000 [1]. This calculation applies to either comparison (A1 vs A0 or B1 vs B0).

The rate of recruitment to the trial depends on the infection rate and so could vary enormously. We therefore measure trial time in number of patients followed to 28 days, rather than in calendar time.

1.2. Two-arm two-stage MAMS design

We now consider a two-stage version of this design under Royston et al.'s MAMS framework [2]. This approach has been extended to trials with binary outcomes [3]. The nstagebin Stata command has been developed to calculate the stagewise sample sizes and trial timelines for trial with binary outcomes [4]. We again consider a single comparison.

Table 1 below presents the design parameters and the operating characteristics for this two-stage trial. The overall type I error rate and power the trial is similar to those of the conventional two-arm design. The two-stage design includes an interim lack-of-benefit analysis on the same primary outcome at 50% of information time, i.e. with a total sample size of 1500.

In this design, we use high (design) power in the interim stage (e.g. recommended to be at least 0.95) to give an effective intervention arm a stronger chance of reaching the final stage, thus allowing more data to be collected on it. For this reason, a design power of 98.5% is chosen for the interim lack-of-benefit analysis. Furthermore, an interim stage

significance level of 0.45 is chosen for stage 1 which acts as the stopping boundary for the lack-of-benefit analysis, which allows us to conduct the stage 1 analysis at 50% of information time when we have 1500 individuals with complete outcome – see also the nstagebin Stata code and output below. The design has a maximum sample size of 2996, very similar to the two-arm conventional design, and expected sample sizes of 2284 and 2977 under the null and alternative hypothesis, respectively. If one is also interested in stopping for overwhelming efficacy at the interim stage, Haybittle-Peto (HP) stopping boundaries can easily be included in the design with negligible impact on the design operating characteristics and trial timelines.

Table 1: Design parameters for a two-arm two-stage African COVID-19 trial.

**Design parameters**	**Stage 1**	**Stage 2**
Primary outcome	28-day mortality	28-day mortality
Control arm event rate	16.73%	16.73%
Intervention effect under H0	0	0
Intervention effect under H1	-4.18%	-4.18%
Allocation ratio	1	1
Power	0.985	0.90
Significance level (one-sided)	0.45	0.025
Sample size	1502	2996
Accrual rate*	50 / week	50 / week
Follow-up	4 weeks	4 weeks
Trial timelines	34 weeks	64 weeks

* This is the (assumed) average accrual rate in each stage. It was used to calculate trial timelines.

The nstagebin Stata code and output are included below.

```
. nstagebin, nstage(2) arms(2 2) alpha(0.45 0.025) power(0.985 0.90)
theta0(0) theta1(-0.0418) ctrlp(0.1673) accrate(50 50) aratio(1) tunit(5)
fu(4) ess

n-stage trial design                 version 1.0.1, 17 Jul 2014
---------------------------------------------------------------
Sample size for a 2-arm 2-stage trial with binary outcome based
on Bratton et al. (2013) BMC Med Res Meth 13:139
---------------------------------------------------------------

Control arm event rate = 0.17
Delay in observing outcome = 4 weeks

Operating characteristics
----------------------------------------------------------------------
```

	Alpha(1S)	Power	theta|H0	theta|H1	Length*	Time*
Stage 1	0.4500	0.985	0.000	-0.042	34.040	34.040
Stage 2	0.0250	0.900	0.000	-0.042	29.880	63.920
Pairwise	0.0245	0.896				63.920

* Length (duration of each stage) is expressed in week periods

Cumulative sample sizes per arm per stage

	Stage 1			Stage 2		
	Overall	Control	Exper.	Overall	Control	Exper.
Number of active arms	2	1	1	2	1	1
Accrual rate*	50.0	25.0	25.0	50.0	25.0	25.0
Patients for analysis	1502	751	751	2996	1498	1498
Patients recruited**	1702	851	851	2996	1498	1498

* Accrual rates are specified in number of patients per week
** Accounts for loss-to-follow-up rate and includes those recruited during follow-up periods

Expected sample size | 0 effective arms = 2284
Expected sample size | 1 effective arms = 2977

1.3. <u>Factorial-MAMS design</u>

We now further extend the above calculations to allow for the factorial nature.

If we allow for the other intervention being effective with the target intervention effect δ, then the control mortality is reduced from π_0 to $\pi_0(1-\delta)$ in patients receiving the effective intervention, so that the overall control mortality for the comparson of interest is the avearge of these, $\pi_0(1-\delta/2) = 14.64\%$. This gives an increased sample size requirement of 3502 to retain 90% power. We choose instead to keep the sample size of 3000 and observe that the power will be reduced from 90% to 85.1% if the other intervention is effective.

Now suppose that an intervention A2 is added when half the planned patients (1500) have been randomised, as in Figure 2D and Table 2 (end of stage 1). We assume we continue with 1: 1: 1 randomisation, because the optimal allocation in the ratio $\sqrt{2}$: 1: 1 gains only some 3% efficiency. This has consequences for each comparison.

- For the A1 vs A0 comparison, it means that only two thirds of randomised patients contribute to this comparison. So this comparison will be mature when $1500 + 1500 * \frac{3}{2} = 3750$ patients have been followed to 28 days (Table 2, end of stage 3).

- At this point, 1500 patients contribute to the A2 vs A0 comparison. This comparison will therefore be mature when a further 1500 patients have been followed, making a total of $1500 + 1500 * \frac{3}{2} + 1500 = 5250$ patients followed (Table 2, end of stage 4).
- For the B1 vs B0 comparison, the only impact is if A2 is an effective intervention. If both A1 and A2 are effective with the target intervention effect δ, then we have to change the control mortality to $\pi_0(1 + 2\delta)/3 = 13.94\%$. This suggests that the power will be further reduced to 83.1%, a loss of power that occurs only if two other interventions are effective and would therefore probably be found acceptable.

The loss of power associated with other intervention being effective suggests that a larger initial power should be chosen, perhaps 90% rather than 80%.

2. Analysis in a single statistical model

We describe how the stage and interventions could be coded in order to estimate all the intervention effects simultaneously, using the trial in Figure 2D as an example.

We first consider the case where all patients are eligible for all interventions. The table lists the possible configurations of randomisations A and B, and NR denotes “not randomised”. The analysis model includes the variables in the final three columns (Stage, A and B) as factor variables. By including a main effect of stage, the model allows patients recruited to each stage to have different prognoses; by not including an interaction between stage and interventions A and B, it assumes the intervention effects for A and B are the same across stages. The coding of B as 9 in stages 3 and 4 is arbitrary, and any value other than 0 or 1 could have been used.

Stage	Randomisations				Coding		
	A options	B options	A allocation	B allocation	Stage variable	A variable	B variable
1	0,1	0,1	0	0	1	0	0
	0,1	0,1	0	1	1	0	1
	0,1	0,1	1	0	1	1	0
	0,1	0,1	1	1	1	1	1
2	0,1,2	0,1	0	0	2	0	0
	0,1,2	0,1	0	1	2	0	1
	0,1,2	0,1	1	0	2	1	0

	0,1,2	0,1	1	1	2	1	1
	0,1,2	0,1	2	0	2	2	0
	0,1,2	0,1	2	1	2	2	1
3	0,1,2	NR	0	NR	3	0	9
	0,1,2	NR	1	NR	3	1	9
	0,1,2	NR	2	NR	3	2	9
4	0,2	NR	0	NR	4	0	9
	0,2	NR	2	NR	4	2	9

Analysis proceeds using a suitable regression model (here, a logistic model) with Stage, A and B as factor variables. Interest lies in the effects of A = 1 and 2 compared with 0 and B = 1 compared with 0. The intervention effect estimands in the all-in-one analysis model are conditional on stage and other interventions and are therefore not strictly comparable with the unconditional estimands in the simpler analysis if the statistical model is non-collapsible [5], but differences are typically small.

We now consider the more complex case where some patients are ineligible for some interventions. In the table below, thicker lines separate groups with the same randomisation options / ineligibilities, which are used to define substages. Substages are equivalent to the interaction of A and B options. The use of the value 9 to code "NR" is again arbitrary and any value could be used. The analysis model again includes the variables in the final three columns (Substage, A and B) as factor variables: this means that we are allowing a different parameter for every substage. The coefficients of substage and of intervention=9 are nuisance parameters of no clinical interest. The model as written contains too many nuisance parameters, some of which will therefore be dropped by the statistical package: for example, the contrast between stages 1.0 and 1.1 is completely confounded with that between B=9 and B=0.

Stage	Randomisations				Coding		
	A options	B options	A allocation	B allocation	Substage variable	A variable	B variable
1	0,1	0,1	0	0	1.0	0	0
	0,1	0,1	0	1	1.0	0	1
	0,1	0,1	1	0	1.0	1	0
	0,1	0,1	1	1	1.0	1	1
	0,1	NR	0	NR	1.1	0	9
	0,1	NR	1	NR	1.1	1	9
	NR	0,1	NR	0	1.2	9	0

	NR	0,1	NR	1	1.2	9	1
2	0,1,2	0,1	0	0	2.0	0	0
	0,1,2	0,1	0	1	2.0	0	1
	0,1,2	0,1	1	0	2.0	1	0
	0,1,2	0,1	1	1	2.0	1	1
	0,1,2	0,1	2	0	2.0	2	0
	0,1,2	0,1	2	1	2.0	2	1
	0,1,2	NR	0	NR	2.1	0	9
	0,1,2	NR	1	NR	2.1	1	9
	0,1,2	NR	2	NR	2.1	2	9
	0,1	0,1	0	0	2.2	0	0
	0,1	0,1	0	1	2.2	0	1
	0,1	0,1	1	0	2.2	1	0
	0,1	0,1	1	1	2.2	1	1
	0,1	NR	0	NR	2.3	0	9
	0,1	NR	1	NR	2.3	1	9
	0,2	0,1	0	0	2.4	0	0
	0,2	0,1	0	1	2.4	0	1
	0,2	0,1	2	0	2.4	2	0
	0,2	0,1	2	1	2.4	2	1
	0,2	NR	0	NR	2.5	0	9
	0,2	NR	2	NR	2.5	2	9
	NR	0,1	NR	0	2.6	9	0
	NR	0,1	NR	1	2.6	9	1
3	0,1,2	NR	0	NR	3.0	0	9
	0,1,2	NR	1	NR	3.0	1	9
	0,1,2	NR	2	NR	3.0	2	9
	0,1	NR	0	NR	3.1	0	9
	0,1	NR	1	NR	3.1	1	9
	0,2	NR	0	NR	3.2	0	9
	0,2	NR	2	NR	3.2	2	9
4	0,2	NR	0	NR	4	0	9
	0,2	NR	2	NR	4	2	9

3. References for supplementary materials

1. Marley-Zagar E, White IR, Royston P, et al. artbin: Extended sample size for randomised trials with binary outcomes. *Stata J*; under review.
2. Royston P, Barthel FM-S, Parmar MK, et al. Designs for clinical trials with time-to-event outcomes based on stopping guidelines for lack of benefit. *Trials* 2011; 12: 81.
3. Bratton DJ, Parmar MKB, Phillips PPJ, et al. Type I error rates of multi-arm multi-stage

clinical trials: strong control and impact of intermediate outcomes. *Trials* 2016; 17: 309.

4. Bratton D. NSTAGEBIN: Stata module to perform sample size calculation for multi-arm multi-stage randomised controlled trials with binary outcomes, https://econpapers.repec.org/RePEc:boc:bocode:s457911 (2014, accessed 13 September 2021).

5. Burgess S. Estimating and contextualizing the attenuation of odds ratios due to non collapsibility. *Commun Stat - Theory Methods* 2017; 46: 786–804.
